# Extensive Genomic Diversity among Bovine-Adapted *Staphylococcus aureus*: Evidence for a Genomic Rearrangement within CC97

**DOI:** 10.1371/journal.pone.0134592

**Published:** 2015-08-28

**Authors:** Kathleen E. Budd, Finola McCoy, Stefan Monecke, Paul Cormican, Jennifer Mitchell, Orla M. Keane

**Affiliations:** 1 Animal & Bioscience Department, AGRIC, Teagasc, Grange, Dunsany, Co. Meath, Ireland; 2 School of Veterinary Medicine, University College Dublin, Belfield, Dublin 4, Ireland; 3 Animal Health Ireland, Carrick-on-Shannon, Co. Leitrim, Ireland; 4 Alere Technologies GmbH, Löbstedter Straße 103–105, D-07749 Jena, Germany; University Medical Center Utrecht, NETHERLANDS

## Abstract

*Staphylococcus aureus* is an important pathogen associated with both human and veterinary disease and is a common cause of bovine mastitis. Genomic heterogeneity exists between *S*. *aureus* strains and has been implicated in the adaptation of specific strains to colonise particular mammalian hosts. Knowledge of the factors required for host specificity and virulence is important for understanding the pathogenesis and management of *S*. *aureus* mastitis. In this study, a panel of mastitis-associated *S*. *aureus* isolates (n = 126) was tested for resistance to antibiotics commonly used to treat mastitis. Over half of the isolates (52%) demonstrated resistance to penicillin and ampicillin but all were susceptible to the other antibiotics tested. *S*. *aureus* isolates were further examined for their clonal diversity by Multi-Locus Sequence Typing (MLST). In total, 18 different sequence types (STs) were identified and eBURST analysis demonstrated that the majority of isolates grouped into clonal complexes CC97, CC151 or sequence type (ST) 136. Analysis of the role of recombination events in determining *S*. *aureus* population structure determined that ST diversification through nucleotide substitutions were more likely to be due to recombination compared to point mutation, with regions of the genome possibly acting as recombination hotspots. DNA microarray analysis revealed a large number of differences amongst *S*. *aureus* STs in their variable genome content, including genes associated with capsule and biofilm formation and adhesion factors. Finally, evidence for a genomic arrangement was observed within isolates from CC97 with the ST71-like subgroup showing evidence of an IS431 insertion element having replaced approximately 30 kb of DNA including the *ica* operon and histidine biosynthesis genes, resulting in histidine auxotrophy. This genomic rearrangement may be responsible for the diversification of ST71 into an emerging bovine adapted subgroup.

## Introduction

Mastitis, which encompasses any inflammatory process that occurs in the mammary gland, is predominantly caused by bacterial infection [1]. Bovine mastitis pathogens are classically referred to as contagious or environmental species depending on their behaviour within dairy herds. Contagious pathogens generally spread from cow to cow with the infected udder being the primary source of infection whereas environmental pathogens, which are found in the environment where the cow resides, spread directly to the udder from the environment [2]. *Staphylococcus aureus*, a major mastitis pathogen is commonly considered a contagious pathogen, although recently it has been recognized that its epidemiological behaviour is not clear cut, with strains demonstrating contagious and/or environmental transmission patterns [3].

*S*. *aureus* presents an important economic problem for the global dairy industry [4, 5] and a poor cure rate has been identified as a significant hurdle for dairy producers [6]. Antimicrobial resistance of *S*. *aureus* is also an increasingly important issue. This bacterium has developed resistance to multiple classes of antibiotics including methicillin and other β-lactams [7, 8] and the horizontal transfer of antimicrobial resistance determinants between livestock and human-associated isolates is an increasing public health concern [9].

Previous studies, which compared diverse strains of *S*. *aureus*, identified genes common to all strains and these comprise the core genome [10, 11]. The remainder of the genome, termed the variable genome, is composed of strain-specific accessory genes often involved in virulence and the ability to colonise specific hosts or environments [12]. Specific lineages are adapted to colonise particular mammalian hosts [12, 13], however, host range barriers are not absolute with some lineages demonstrating a broad host range, while host shifts have also been reported [14]. The ability of *S*. *aureus* to adapt to a specific host is influenced by the acquisition of mobile genetic elements, gene diversification and decay [14]. Understanding the combinations of genes which are responsible for the success of dominant clonal lineages of *S*. *aureus*, and knowledge of the factors required for host specificity and virulence are important for understanding the pathogenesis, management and treatment of *S*. *aureus* mastitis.

In this study, the genetic heterogeneity of 126 *S*. *aureus* isolates from cases of clinical mastitis in Ireland was evaluated both at the core and variable genome levels. The clonality of the isolates, the presence of important genes associated with virulence and the antimicrobial susceptibility of the isolates to antibiotics commonly used to treat mastitis was also determined. Finally a genomic rearrangement in a subgroup of isolates belonging to clonal complex (CC) 97 was characterised.

## Materials and Methods

### Bacterial isolates

All 126 *S*. *aureus* isolates used in this study were recovered from milk samples taken from cows presenting clinical mastitis between February 2010 and February 2011 from 26 farms in Ireland. Sample collection and bacterial isolation methods have been described previously [15]. Staphylococci were identified based on colony morphology, Gram stain, haemolysis, catalase test and growth on Baird Parker and Mannitol Salt agar plates. Putative *S*. *aureus* were distinguished from coagulase negative Staphylococci by the above tests as well as the coagulase test and the API Staph strip (BioMerieux). Isolates were routinely grown in Trypticase Soy broth (TSB) or on Trypticase Soy agar for further study.

### Antimicrobial susceptibility testing

Antimicrobial susceptibility testing was carried out on all isolates using the disk diffusion method in accordance with the Clinical and Laboratory Standards Institute (CLSI) guidelines [16]. Penicillin (6 μg/10 IU), ampicillin (10 μg), amoxycillin and clavulanic acid (20 μg + 10 μg), oxacillin (1 μg), tetracycline (30 μg), kanamycin (30 μg), neomycin (30 IU), ceftiofur (30 μg), enrofloxacin (5 μg), erythromycin (15 μg), clindamycin (2 μg) cefalexin (30 μg) and vancomycin (30 μg) were tested, with *S*. *aureus* ATCC 25923 (penicillin susceptible) and *E*. *coli* ATCC 25922 (penicillin resistant) acting as control strains. Isolates were considered resistant, intermediate or susceptible based on the diameter of their zone of inhibition when compared with the zone diameter interpretive standards for staphylococcal veterinary pathogens [16]. Isolates classified as intermediate were considered susceptible for analysis purposes. There are currently no CLSI breakpoints approved to indicate neomycin resistance in veterinary Staphylococci and so a resistant zone diameter breakpoint of ≤ 16 mm for neomycin was used [17]. Oxacillin was used for the detection of methicillin resistance.

### Genomic DNA extraction

Genomic DNA was extracted from 2 mL of an overnight culture in TSB using the PurElute Bacterial Genomic Kit (Edge Biosystems, MD, USA) as described in the manufacturer’s protocol with the exception of the addition of 100 μg/mL lysostaphin (AMBI products LLC, NY, USA) to the Spheroblast lysis buffer followed by incubation at 37°C for 1 h. DNA pellets were resuspended in 100 μl distilled H_2_O and stored at -20°C until use. DNA concentration was determined using a nanodrop ND-1000 spectrophotometer by measuring the absorbance of the sample at a wavelength of 260 nm. For next generation sequencing, genomic DNA was further purified using the DNA clean and concentrator kit (Zymo Research, USA) before quantification using the Qubit DNA quantification kit (Life Technologies, Ger).

### Multi locus sequence typing

Multi-Locus Sequence Typing was performed according to the methods described on the *S*. *aureus* MLST database (http://saureus.mlst.net/) and by Enright *et al*., [18]. PCR products were purified with the QIAamp PCR purification kit (Qiagen) and sent to Beckman Coulter Genomics (Essex, UK) or Source Bioscience Genomics Service (Dublin, Ireland) for Sanger Sequencing with the forward and reverse amplification primers. Sequence chromatograms were checked for quality and trimmed using Bioedit V7.0.0 (http://www.mbio.ncsu.edu/bioedit/bioedit.html). Alleles and STs for each isolate were assigned using the MLST database (http://saureus.mlst.net/). The MLST data set was subdivided into non-overlapping groups of related STs or CCs and a founding genotype for that complex predicted using the eBURST algorithm V3 [19]. Related genotypes were defined as those where all members assigned to the same group share identical alleles at ≥ six of the seven loci with at least one other member of the group. In some cases, a single locus variant (SLV) of the primary founder may diversify to produce multiple SLVs and this was denoted a subgroup founder.

### Population genetics and phylogenetic analyses

The population-scaled mutation and recombination parameters were estimated using ClonalFrame V1.2 [20]. The concatenated nucleotide sequence of each of the seven MLST loci were used as input and a single representative of each ST was used in order to minimize bias resulting from the overrepresentation of particular clones. In total, 5 independent runs of the Monte Carlo Markov Chain were performed with 100,000 burn-in iterations and a posterior sampling of 200,000 iterations. The burn-in iterations were discarded and model parameters were estimated every 100 iterations from the posterior. Satisfactory convergence of the Markov chain in the different runs was estimated using the Gelman-Rubin statistic, which was ≤ 1.2 for all parameters indicating convergence of the Markov chain. To test the role of recombination in generating allelic variation, the pairwise homoplasy index (PHI) test [21], implemented in SplitsTree v4.0 software [22], was calculated for each locus.

### Microarray genotyping

Sensitive and specific miniaturised microarrays (Alere technologies, Jena, Germany) encoding gene targets for the identification of *S*. *aureus* virulence genes, antimicrobial resistance genes, and species markers were used for genetic characterization of the *S*. *aureus* isolates. Details of the array have been reported previously [23]. Briefly, DNA was purified using the DNeasy Blood and Tissue kit (Qiagen, Germany), amplified and labelled in a multiplex primer elongation reaction, hybridized to the array and finally a horseradish-peroxidase-streptavidin triggered dye precipitation reaction resulted in formation of visible spots in the case of a positive reaction. The array includes 333 target sequences that equates to approximately 170 distinct genes and their allelic variants. Details of the hydridisation profiles of individual isolates are available in the S1 Table.

### Cluster dendrogram construction

Phylogenetic-like analysis of the microarray hybridization pattern profile was performed using R (V3.0.2, http://r-project.org). Genes were denoted as detected (‘1’), not detected (‘0’) or ambiguous (‘NA’) in each sample. The Euclidean distance matrix was computed to measure the similarity of gene hybridization profiles amongst the isolates using the dist function in the package “Stats”. The cluster dendrogram was generated using the hierarchical agglomerative clustering method and the hclust function in “Stats” that is based on Ward’s method [24]. The hierarchical clustering was confirmed via multiscale bootstrap resampling using the pvclust package [25] in R, with p-values computed for all clusters.

### Whole genome sequencing, assembly and annotation

Paired-end sequencing libraries were constructed by sonicating 2.5 μg of genomic DNA in 55 μL of buffer EB (Qiagen, Germany) using a bioruptor (Diagenode) for 30 seconds on, 90 seconds off for a total sonication time of 10 minutes for a target fragment size of 550 bp. Fragments were end-repaired, size selected, A-tailed and Illumina adapters ligated according to the Illumina TruSeq DNA PCR-free sample preparation kit instructions. The libraries were quantified using the Illumina SYBR universal library quantification kit (Kapa biosystems, USA) and pooled before sequencing using an Illumina MiSeq generating 300 bp paired-end reads. Sequence quality assessment and filtering was carried out using FASTQC V0.10.1 (http://www.bioinformatics.babraham.ac.uk/projects/fastqc/) and Trimmomatic V0.30 softwares [26]. Briefly, Illumina fastq files were quality filtered to remove/trim reads containing sequencing adaptor read-through, a median quality score below 20 and more than 2 uncalled bases. Following quality control, an average of 745,000 (+/- 128,000) paired end reads per strain were carried forward for assembly. For each sequenced strain a draft genome assembly was generated in two separate steps. IDBA-UD V1.1.1 [27] was first used to assemble the raw reads into contigs. SSPACE V1.1 [28] was subsequently used to scaffold the pre-assembled contigs, based on paired-end reads mapping to the edges of these contigs. The quality of each assembly was assessed using QUAST V2.3 [29] and the reference sequence *Staphylococcus aureus* subsp. *aureus* NCTC 8325. The draft assembled genomes for each strain were annotated with the RAST annotation system [30] using FigFAM release 70 and GLIMMER V3.02 [31]. Sequences were deposited in the NCBI Sequence Read Archive with accession number SRP050409.

### Biofilm formation

Isolates were grown overnight in TSB at 37°C with shaking before being diluted 1:100 in TSB, TSB + 1% glucose or TSB + 4% NaCl and 100 μL transferred to each of four wells of a sterile non-tissue culture treated U bottom polystyrene plate (Sarstedt, Germany). The plates were incubated statically for 24 h. The supernatant was then discarded and plates washed three times in water to remove planktonic bacteria. Crystal violet solution (100 μL) was added for 10 min. The plates were again washed in water and allowed to dry before the addition of 200 μL of 30% (w/v) acetic acid and the OD of each well measured at 570 nm. Assays were carried out in triplicate with strain SH1000 used as an inter-plate control. Isolates were classified as no, weak, moderate or strong biofilm producers as previously described [32]. Due to the low number of strong biofilm producers, data for moderate and strong biofilm producers was combined. Association between biofilm production and genotype was assessed using the Freeman-Halton extension of Fishers’ exact test.

### Histidine auxotrophy

A synthetic minimal medium with and without histidine was used to determine histidine auxotrophy. This medium was adapted from [33] with the following modifications i) 5.8 mg/L FeCl_3_.6H_2_O replaced 6 mg/L FeSO_4._7H_2_O, ii) 5.65 mg/L MnCl_2._4H_2_O replaced 10 mg/L MnSO_4._4H_2_O iii) amino acids were present at a concentration of 250 mg/L.

## Results

### MLST and eBURST

From 126 *S*. *aureus* isolates from 26 farms, 18 different STs were identified. The most common STs were ST151 (n = 32) and ST71 (n = 27) followed by ST136 (n = 14) and a novel ST, ST3170 (n = 13). Five STs were observed only once in the dataset and the dataset contained eight novel STs not previously deposited in the MLST database. Novel STs were based on either the presence of an allele not annotated in the MLST database (7/8, 88%) or a unique combination of known alleles (1/8, 12%). The number of alleles per locus varied from 4 (*pta* and *tpi*) to 9 (*yqiL*) while the number of polymorphic sites per locus varied from 6 (*arcC*) to 23 (*yqiL*). The *yqiL* gene fragment had many more polymorphic sites than any other gene fragment. MLST results are shown in Table 1. For 19 of the 26 farms, more than one *S*. *aureus* isolate was recovered. On a number of farms more than one STs was isolated indicating there may be multiple reservoirs of infection on these farms (Table 2).

**Table 1:** Number of isolates, number of herds in which it was detected and clonal complex affiliation for each Sequence Type (ST).

ST	No. of isolates	No. of herds	Clonal complex
1	3	3	1
5	2	1	5
71	27	10	97
97	5	2	97
136	14	2	ST136
151	32	15	151
1074	4	3	151
1123	2	2	151
1278	1	1	1
3085	1	1	97
3170*	13	2	97
3171*	3	1	151
3172*	3	2	97
3173*	10	1	97
3174*	1	1	151
3175*	1	1	97
3219*	3	1	97
3221*	1	1	97

*Denotes novel ST submitted to the MLST database

**Table 2:** Total number of *S*. *aureus* isolates and number of unique Sequence Types (STs) from each farm where >1 *S*. *aureus* was isolated.

Farm Number	Total number of *S*. *aureus* isolates	Number of unique STs
1	18	3
2	3	3
3	7	5
4	6	3
5	5	2
6	17	5
7	15	4
8	14	2
9	4	3
10	3	2
11	2	1
12	3	1
14	2	1
15	6	3
16	2	1
19	3	1
20	3	1
22	3	1
23	3	1

**Fig 1:**
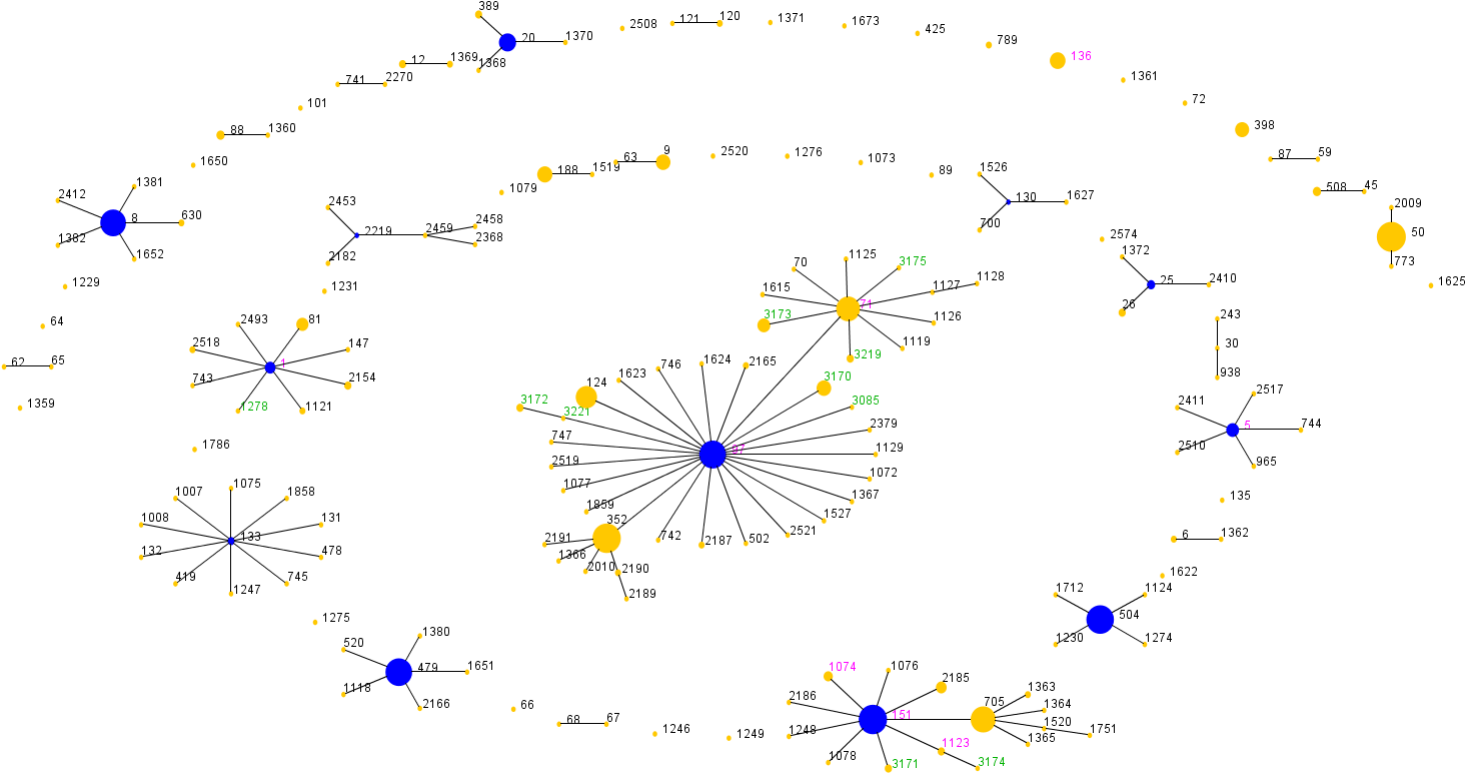
eBURST analysis of mastitis-associated *S*. *aureus*. Sequence types (STs) from this study were compared to all STs of bovine origin in the MLST database. Circles represent different STs; blue represents a primary founder, yellow a subgroup founder, pink denotes STs found in both datasets, green STs found in present study dataset only and black STs in the MLST database dataset only. All lines represent a single locus difference and the size of the circles is relative to the number of isolates.

Groups of related genotypes were identified using the eBURST algorithm. The isolates in this study were compared with all entries of bovine origin in the MLST database. Despite the genetic heterogeneity among the isolates, all grouped into CCs/STs (Fig 1), which are previously reported to be bovine associated [34]. The majority of isolates grouped into CC97 (n = 64), CC151 (n = 42), CC1 (n = 4) and CC5 (n = 2) with the remaining 14 isolates belonging to ST136. The predicted founders of all four CCs identified were present amongst the STs. Assignment of the ancestral founder was supported by the eBURST analysis with bootstrap resampling yielding values of 99%, 85% 99% and 97% for the prediction of the respective founding STs. Noticeably, ST71 appears to have formed a subgroup within CC97 and a number of the CC97 isolates were more closely related to ST71 than ST97.

### Population genetics and phylogenetic analyses

To examine the role of recombination on *S*. *aureus* population structure, the concatenated MLST nucleotide sequences were analysed using ClonalFrame. The mean recombination tract length (Δ) was estimated to be 152 bp (95% Confidence Interval 11–850 bp). The estimate of the rate at which nucleotides change by recombination versus point mutations (r/m) was 1.38 (95% CI 0.19–5.49) suggesting that a nucleotide substitution was more likely to be due to recombination compared to point mutation. The data was further analysed with SplitsTree. The PHI test detected a potential signature of recombination within the *yqiL* locus (P = 0.078) but at no other locus (P = 1.0).

### Antimicrobial susceptibility testing

Isolates were tested for resistance to antibiotics commonly used to treat mastitis. The results of the antimicrobial susceptibility testing are shown in S2 Table. Approximately half of the isolates (52%) were resistant to penicillin and ampicillin but the isolates were susceptible to the other antibiotics tested. Multi-drug resistance, defined as resistance to three or more antibiotics, was not observed. Penicillin and ampicillin resistance were significantly associated with CC affiliation. All isolates of CC1 and CC151 were penicillin and ampicillin susceptible and ST136 was predominantly susceptible (92%). The two isolates belonging to CC5 were resistant while isolates belonging to CC97 were predominantly resistant (98%). No isolate displayed oxacillin resistance despite isolates from CC1 and CC5 being associated with MRSA in previous reports [35, 36].

### DNA-microarray based genotyping

The microarray evaluation software (Iconoclust, Alere Technologies) assigns isolates to CCs based on their hybridization profile. All isolates were assigned to the CCs identified through MLST. Within CC97 2 subgroups with different array hybridisation profiles were noted, a subgroup which included ST97 (denoted as typical CC97) and a subgroup which included ST71 (denoted as ST71-like). It was found that one ST (ST3085) was assigned to CC97 by MLST but the ST71-like subgroup according to the array hybridization profile. This isolate was a single locus variant of both ST97 and ST71 but appeared to be more similar to ST71 group when its variable genome was taken into account. CCs mentioned hereafter refer to those assigned based on microarray genotyping and so there was typical CC97 (n = 22), subgroup ST71 (n = 42), CC151 (n = 42), ST136 (n = 14), CC1 (n = 4) and CC5 (n = 2).

#### Antimicrobial resistance genes

Components of the β-lactamase operon (*blaZ*, *blaI*, *blaR*) were found in 67 (53%) of the isolates, 66 of which were phenotypically penicillin and ampicillin resistant. All CC97 and CC5 isolates indicated presence of *bla* genes in addition to one isolate from ST136. One isolate from the ST71-like subgroup of CC97 encoded the *bla* genes but was phenotypically susceptible. A small number of isolates (n = 4) belonging to CC97 gave ambiguous results for *blaI* or *blaR* but all contained *blaZ* and all were phenotypically resistant. No components of the *mec* operon, responsible for methicillin resistance were detected and no isolate was phenotypically resistant. All isolates carried an unspecific efflux pump gene (*sdrM*). One isolate from CC97 was positive for *ermC*, associated with macrolide and lincosamide resistance; however this isolate was not phenotypically resistant to the macrolide or lincosamide tested. The probe for *vanB* yielded signals in two isolates belonging to CC97 but these isolates were not phenotypically resistant.

#### Agr-typing

All CC5 and CC151 isolates encoded the accessory gene regulator type II (*agr*II). Isolates of both subgroups of CC97 encoded *agr*I which was expected given the relatedness of these two subgroups. The bovine associated ST136 and human-associated CC1 isolates were associated with *agr*III.

#### Capsule and biofilm associated genes

The majority of isolates were *cap8* positive (n = 88) with the remaining isolates encoding *cap5* (n = 38). Despite the evolutionary relationship between typical CC97 and the ST71-like subgroup they encoded different capsule types with typical CC97 encoding *cap5* and ST71-like encoding *cap8*. CC151 and CC1 also encoded *cap8* while CC5 and ST136 were capsule type 5. All isolates with the exception of the ST71-like sub-group, were positive for *icaA*, *icaC* and *icaD*, components of the *ica* operon responsible for the production of extracellular poly-N-acetylglucosamine (PNAG) which facilitates biofilm formation. Some ST71-like isolates (n = 12) were positive for *icaA* but not *icaC* or *icaD*. All isolates were negative for the *bap* gene, which encodes a biofilm associated protein involved in the production of a proteinaceous biofilm matrix.

#### Virulence genes

All isolates were negative for genes encoding the Panton-Valentine leukocidin (*lukS/F-PVL*) although isolates belonging to CC151 were positive for *lukF-P83* and *lukM*, the Panton Valentine-like leukocidin F and S components from ruminant-adapted strains of *S*. *aureus*. The genetically linked leukocidin components, *lukF and lukS* as well as *lukD* and *lukE* were found in all isolates with the exception that signals for *lukF* and *lukE* were not detected from one isolate from the ST71-like group and a *lukE* signal was not detected in seven isolates from ST136. However, absence of these genes would need to be confirmed by genome sequencing as absence of a signal may be due to allelic variation resulting in poor hybridisation. Among the haemolysin gene family, high abundance was detected across all complexes for *hla*, *hlb*, *hld* and *hlIII*. However, CC151, ST136 and one ST71-like isolate encoded an alternative RF122-like allele of *hlIII* compared to the other complexes. The human-specific immune evasion cluster of *sak* (staphylokinase), *chp* (chemotaxis-inhibiting protein) and *scn* (staphylococcal complement inhibitor) was not detected in any complex with the exception of CC5 and these isolates showed a disrupted *hlb* gene. CC5 has a broad host range and both CC5 isolates were recovered from the same farm and may represent local human-animal transmission. Signals for exfoliative toxin genes *etA*, *etB*, *etD*, the genes encoding epidermal cell differentiation inhibitors *edinA*, *edinB*, *edinC* and the ACME cluster were also not detected in any isolate.

The gene *tst1*, which encodes toxic shock syndrome toxin, was observed in 30 isolates, all of which belonged to CC151. All isolates belonging to CC151 and the ST71-like subgroup of CC97 were also positive for *orfCM14*, an enterotoxin homologue (GenBank U10927.2 [32627 to 33406]). It is important to note that *orfCM14* was the only toxin-related gene present amongst ST71-like isolates and it was not detected in typical CC97 isolates. CC151 and CC5 isolates all contained the enterotoxin coding genes *seg*, *sei*, *sem*, *sen*, *seo* and *seu* encoded on *egc*, the enterotoxin gene cluster. In addition to the *egc-*encoded genes, some isolates from CC151 also contained *seb* (n = 7) while those that encoded *tst1* also encoded *sec* and *sel*. CC5 isolates, as well as harbouring *egc*, were positive for *sea*, *seb*, *sed*, *sej*, *sek*, *seq* and *ser*. One ST136 isolate and one CC151 isolate contained *sed* while all CC1 isolates encoded *seh*. Typical CC97 (n = 22, 100%) and ST136 (n = 13, 93%) did not harbour any enterotoxin-associated genes. All complexes possessed a range of superantigen genes including *ssl01*/*set6*, *ssl02*/*set7*, *ssl03*/*set8*, *ssl05*/*set3*, *ssl07*/*set1* and *ssl08*/*set12*. However, the gene *ssl11/set2* was not detected in any isolate belonging to CC97 or ST136.

Adhesion factors and genes encoding microbial surface components recognizing adhesive matrix molecules (MSCRAMMS) were found abundantly in all isolates. The genes *clfA* (clumping factor A), *clfB* (clumping factor B) and *vwb* (von Willebrand factor binding protein) were detected in all isolates, with the bovine adapted CC97 and CC151 carrying an RF122-like allele of *vwb*. Bone sialoprotein-binding protein (*bbp*) was absent from 5 isolates and *fib* (fibrinogen binding protein) and *fnbA* (fibronectin binding protein A) were found in all but one isolate. The *sasG* gene was found in all isolates except those belonging to CC151 while *fnbB* was found in all isolates with the exception of those belonging to CC151 where it was only found in seven (17%) of the isolates. The collagen-binding adhesin *cna* gene was detected in all CC1 isolates and in the majority of the ST71-like subgroup of CC97 (95%). The presence of *cna* in the ST71-like subgroup contrasted with typical CC97 which did not encode *cna*.

### Hierarchical cluster dendrogram

**Fig 2:**
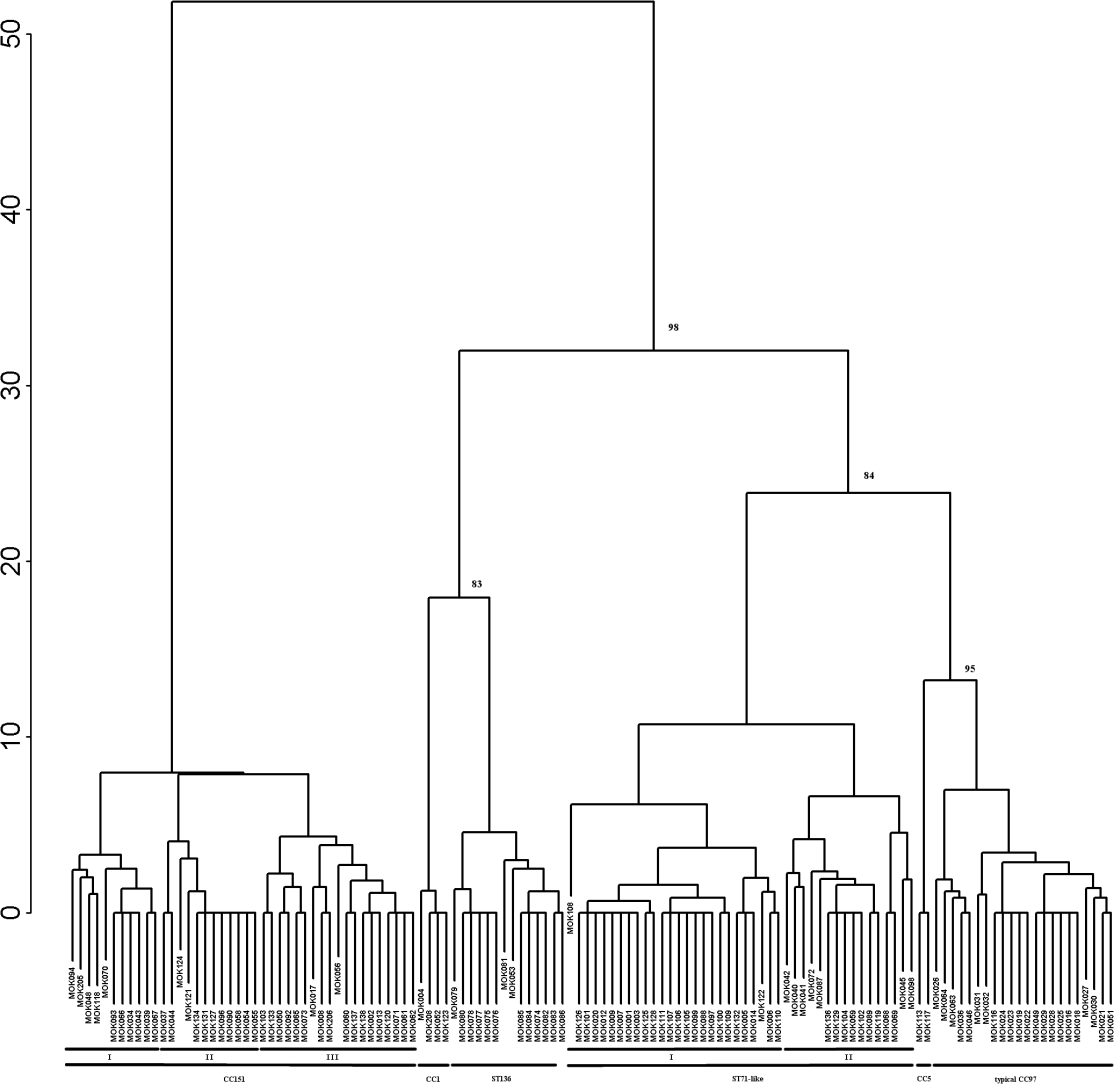
Relationship among the *S*. *aureus* isolates. Hierarchical cluster dendrogram of all isolates based on their hybridization profile from the genotyping array. Bootstrap support values indicated for the major internal branches that separate the clonal complexes. Subgroups within the ST71-like group and CC151 are noted.

Hierarchical cluster analysis based on the array genotyping data grouped isolates according to their CC. Based on the clustering, a number of subgroups within CC151 and the ST71-like subgroup were observed and are shown in Fig 2. The ST71-like isolates formed a separate cluster to the typical CC97 isolates. Hybridization profiles for the ST71-like subgroup and the typical CC97 show a number of gene differences. The ST71-like isolates all possess *orfCM14*, all lack *icaC* and *icaD* (with many also lacking *icaA*) and 95% possess *cna*. This compares to typical CC97 where *ica* is present but *orfCM14* and *cna* are absent. ST71-like isolates are also capsule type 8 whereas typical CC97 is capsule type 5.

Two subgroups were also identified within the ST71-like subgroup by the clustering method and gene differences between these groups include different allele possession for *ssl01/set6* and *ssl6* was not detected in one group. Three subgroups were also identified within CC151, which reflected different *tst* and enterotoxin gene content.

### Typical CC97 and the ST71-like subgroup

**Fig 3:**
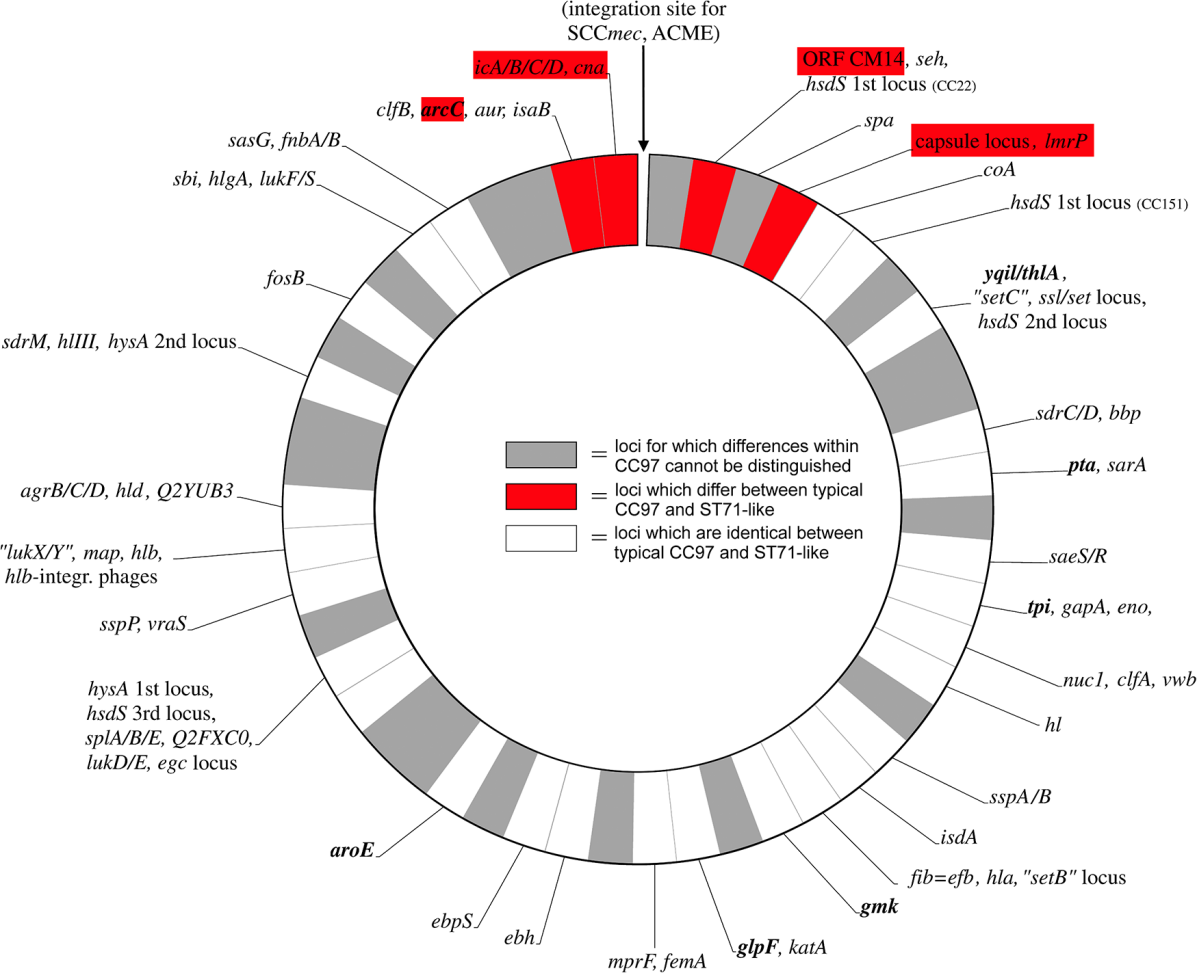
Gene differences for ST97 and ST71. Schematic diagram of *S*. *aureus* genome showing gene differences for the closely related isolates ST97 and ST71.

There were a number of differences in gene content in the region around the origin of replication for the clonally related isolates in the ST71-like subgroup and typical CC97 (Fig 3). These differences are summarised in Table 3. In order to determine if a genomic rearrangement in this region gave rise to these differences eight isolates were subjected to whole genome sequencing, five from typical CC97 and three from the ST71-like subgroup of CC97. The genomes were *de novo* assembled into between 19 and 70 scaffolds with assembly statistics for each isolate in S3 Table. As genes between *coA* and *clfB* did not differ between typical CC97 and the ST71-like subgroup, single contigs containing both *clfB* and *coa* were identified for six isolates and the region from the start of *clfB* to the end of *coa* was extracted. Open reading frames (ORFs) were identified and all annotated genes between *clfB* and *coa* extracted. The length of this region averaged 319,983 bp (range 319,127 bp– 320,202 bp) for typical CC97 isolates and 289,484 bp (range 287,629 bp– 290,529 bp) for the ST71-like isolates indicating that the ST71-like isolates had lost over 30 kb of DNA in this region. A total of 269 ORFs were identified in typical CC97 isolates compared to 234 in the ST71-like isolates (S4 Table). A major genomic rearrangement between the groups occurred downstream of the *sasA* gene. ST71-like isolates appear to have lost almost 30 kb downstream of this gene including the *ica* operon and histidine biosynthesis genes and in their place acquired a IS431 insertion element encoding a typical plasmidic toxin/antitoxin system and two putative exported virulence factors. A number of additional small rearrangements were also detected between typical CC97 and the ST71-like subgroup, most notably the loss of a number of Type I restriction modification system genes by the ST71-like subgroup (S4 Table).

**Table 3:** Genetic differences between typical CC97 (ST97-like) and ST71-like subgroups of CC97.

Gene	ST97-like	ST71-like
*arcC*	Allele 3	Allele 18
*ica* operon	Present	Predominantly Absent
*cna*	Absent	Predominantly Present
*orfCM14*	Absent	Present
Capsule type	*cap5*	*cap8*
*lmrP*	Non-RF122 allele	RF122 allele

Typical CC97 and ST71-like isolates were compared for their ability to form a biofilm in a variety of media. There was isolate-to-isolate variation in biofilm formation (S2 Table) with high osmolarity significantly repressing biofilm formation in both typical CC97 and the ST71-like sub-group (P < 0.001). However, despite the loss of the *ica* operon by the ST71-like isolates there was no significant difference between typical CC97 isolates and ST71-like isolates in their ability to form a biofilm in TSB (P = 0.88) or TSB + 4% NaCl (P = 1) while the difference in TSB + 1% glucose approached significance (P = 0.07). Both subgroups of CC97 were also compared for their ability to synthesise histidine. The loss of the histidine biosynthesis genes resulted in ST71 being auxotrophic for histidine (S1 Fig) unlike typical CC97.

## Discussion

In this study we analysed the clonal diversity of 126 *S*. *aureus* isolates from cases of bovine clinical mastitis in Ireland and determined their virulence gene profile and antimicrobial susceptibility status. A diversity of STs was observed amongst the *S*. *aureus* isolates, with multiple STs found on a number of farms and STs common to multiple herds identified. To-date, control strategies for *S aureus* mastitis have focused on control of contagious mastitis. The results presented here do not entirely support the hypothesis that a predominant strain of *S*. *aureus* exists within a herd and that this strain is likely to be restricted to a single herd [37–39]. The presence of multiple *S*. *aureus* genotypes on many of the farms indicates the presence of multiple reservoirs of infection on these farms and may suggest an environmental transmission pattern. Additional work is warranted to identify major reservoirs of *S*. *aureus* on-farm.

Genetic variation of human-associated *S*. *aureus* has been reported to occur primarily by point mutation [39–41] with a limited role for recombination as it was found that alleles are up to 15-fold more likely to change by point mutation than by recombination [39–42]. However, a more recent study found evidence of widespread core genome recombination across the *S*. *aureus* species [43]. Analysis of our bovine-adapted *S*. *aureus* ST diversification found that a nucleotide substitution was more likely to be due to recombination compared to point mutation. Examination of genetic variation at individual MLST loci revealed that *yqiL* had many more polymorphic sites when compared to the other 6 gene fragments and there was suggestive evidence of a signature of recombination within the *yqiL* locus. The presence of core genome recombination hotspots in the *S*. *aureus* genome has been reported to be associated with the presence of mobile genetic elements [43]. Therefore, one possible explanation for the detection of a recombination signature at the *yqiL* locus is that it is located near the insertion site of a mobile genetic element, although it was not located in a region of the genome with an excess of homoplasy as reported by Everitt *et al*. Evidence of recombination playing an important role in bovine-associated *S*. *aureus* clonal diversification was previously reported in isolates belonging to CC97 [43]. In this study isolates were equally likely to diversify by recombination as point mutation, as was the case in our study, which may indicate differences in the population structure of bovine-associated *S*. *aureus* compared to human-associated *S*. *aureus*. The presence of alleles that have changed by recombination in multiple isolates suggests they are being maintained in the population, possibly as they confer an increased ability to colonize and infect cows [44].

The hypothesis that relatively few widely distributed CCs of *S*. *aureus* are responsible for the majority of cases of bovine intra-mammary infections [45, 46] were supported in this study. Despite the ST diversity amongst the isolates, only four CCs were detected (CC97, CC151, CC5 and CC1) in addition to one ST (ST136). *S*. *aureus* as a causal bacterium of bovine mastitis in Ireland mainly consist of the bovine-specific lineages, CC97, CC151, and ST136.

The DNA microarray-based analysis revealed a number of differences in the variable genomes between the different ST/CCs in this study. Hierarchical cluster analysis grouped isolates according to their CC. The subgroups observed within CC151 and the ST71-like subgroup of CC97 warrant further investigation as despite these isolates being highly related at the core genome level, distinct differences in their variable genome has resulted in subgroups which may continue to diversify. Widespread core genome recombination has recently been reported across the *S*. *aureus* species. Recombination was found to vary across the genome, peaking at the origin-of-replication [43]. The separation of ST71 and its subsequent evolution into a distinct subgroup within CC97 is possible evidence of *S*. *aureus* evolution by recombination. ST71-like isolates were recovered more commonly than typical CC97 isolates, suggesting that this subgroup may be more successful at infecting the bovine mammary gland. The difference between typical CC97 and ST71-like isolates in gene/allele content of *arcC*, *cna*, *ica*, *orfCM14*, *lmrP* and *cap*, all of which are located close to each other near the origin of replication suggested a genomic rearrangement event had occurred in this region. Similar chromosomal rearrangements have been reported previously in *S*. *aureus* and a contribution to host adaptation and clonal evolution was observed [14, 47]. The genomes of isolates from both sub-groups were sequenced and the region around the origin of replication was examined. ST71-like isolates were found to have lost almost 30 kb of DNA in this region compared to typical CC97. A large rearrangement occurred immediately downstream of the *sasA* gene which resulted in the loss of the *ica* operon and histidine biosynthesis genes. This genomic rearrangement resulted in the inability of ST71 to catalyse the *de novo* biosynthesis of histidine with this ST dependent on an external source of histidine. Despite the absence of the *ica* genes, most ST71-like isolates formed a biofilm in one of the media tested. There was variation among the ST71-like and the typical CC97 isolates in their ability to form a biofilm in different environmental conditions, however, no significant difference between the groups was found although the difference in TSB + 1% glucose approached significance. In *S*. *aureus*, PNAG-independent biofilm mechanisms, mediated by proteins, extracellular DNA and teichoic acids have been widely reported [48] and these may be contributing to biofilm formation in one or both groups. A number of additional smaller genomic rearrangements were also detected between ST71-like and typical CC97, however, further work is required to determine the exact causation of the diversification of ST71.

Analysis of toxin and antibiotic resistance genes revealed evidence of association between certain virulence genes and specific ST/CCs. The *lukF-P83*/*lukM* locus, which is phage borne, encodes a bi-component leukotoxin that is highly active against bovine neutrophils [49–52] and was previously reported to be ubiquitous among CC151 isolates [53, 54]. Similarly, in this study the *lukF-P83*/*lukM* locus was detected only among the isolates belonging to CC151 although it has previously been detected in isolates from livestock-associated genotypes CC479, CC130 and ST522; as well as occasionally in CC30 and CC97 [55]. This locus demonstrates host specificity that ultimately may lead to the evolution of distinct bovine populations of *S*. *aureus*. Allelic variation in the *vwb* gene has also been proposed to play a role in host-specificity [56]. All bovine-adapted complexes, with the exception of ST136, reacted with a bovine-specific *vwb* allele on the array, while the specific *vwb* allele carried by ST136 could not be identified. The enterotoxin gene cluster *egc* was only observed in isolates belonging to CC151 and CC5, which was similar to findings by Jamrozy et al., [54]. In human-adapted *S*. *aureus* the *egc* cluster has been previously reported to be more common in carriage strains than invasive isolates [57] although the relevance of this to bovine disease remains to be determined. Comparison of superantigen profiles revealed that all CC/STs shared similar profiles.

The carriage of antimicrobial resistance genes was not heterogeneous as previously observed by Monecke *et al*., [53] who reported that the presence of antibiotic resistance determinants is largely non-lineage specific due to the promiscuous nature of mobile genetic elements. In the present study, the presence of resistance genes, was mainly associated with specific ST/CCs with CC97 and CC5 both harbouring β-lactam resistance genes. With the exception of penicillin/ampicillin resistance, few antibiotic resistance determinants were present amongst the isolates. The low prevalence of antibiotic resistance genes among *S*. *aureus* of bovine origin was similarly observed by Monecke *et al*., [58] indicating that antimicrobial resistance has not been heavily selected for in this population.

In terms of toxin profiles, the CC151 isolates encoded between 8–13 toxins per isolate. This was substantially more toxin genes than the other bovine-adapted CCs which encoded either no toxin genes or just one toxin. The pathogenicity island SaPIbov, which comprises *tst1*, *sec*, and *sel*, was identified in two of the three CC151 subgroups. Its absence in the closely related isolates of CC151 group II could be due to the mobility of the island as previously observed [10]. However, it must be noted that genes will only be detected if they react with probes on the array and so the possibility of isolates encoding alleles that we did not detect cannot be excluded.

In summary, this analysis has identified significant genotypic diversity among Irish bovine-associated *S*. *aureus* isolates. The major complexes identified were CC97, CC151 and ST136 demonstrating geographical differences in the major genotypes associated with bovine-mastitis [55]. Recombination played a notable role in the diversification of the isolates and microarray analysis revealed a number of interesting differences amongst the *S*. *aureus* STs in both their core and variable genome content. These differences include a genomic arrangement event which occurred near the origin of replication in isolates from CC97 and may be responsible for the expansion of ST71 into a large and distinct subgroup. Further studies are required to precisely elucidate these events and to determine the role they play in host specification and clonal evolution.

## Supporting Information

S1 Fig Growth of ST97 and ST71 on minimal media +/- histidine. (TIFF)

S1 Table Complete hydridisation profiles of the *S*. *aureus* isolates. (XLSX)

S2 Table MLST allele profiles, clonal complex affiliation, antimicrobial susceptibility and biofilm formation of the *S*. *aureus* isolates. (XLSX)

S3 Table Assembly metrics for sequencing libraries from typical CC97 and ST71-like isolates. (DOCX)

S4 Table Open reading frames identified between *clfB* and *coa* in typical CC97 and ST71-like isolates. (XLSX)
