# Supplementary figures and images for: Extensive Genomic Diversity among Bovine-Adapted Staphylococcus aureus: Evidence for a Genomic Rearrangement within CC97
Source: PLoS One. 2015 Aug 28;10(8):e0134592. doi: 10.1371/journal.pone.0134592 (PMC4552844; doi:10.1371/journal.pone.0134592)

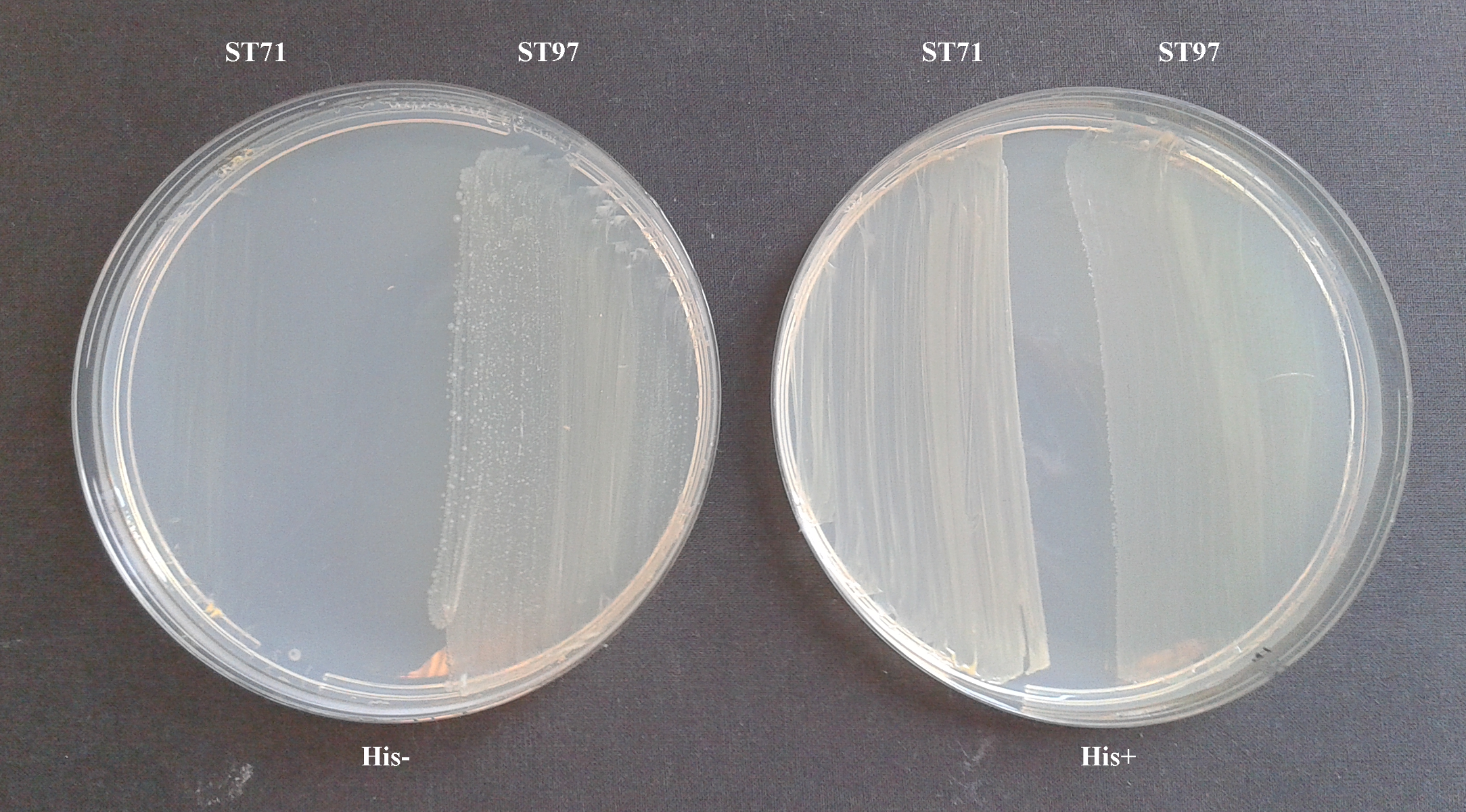

Supplement: S1 Fig — (TIFF) [file pone.0134592.s001.tiff]
