# Supplementary material for: Extensive Genomic Diversity among Bovine-Adapted Staphylococcus aureus: Evidence for a Genomic Rearrangement within CC97
Source: PLoS One. 2015 Aug 28;10(8):e0134592. doi: 10.1371/journal.pone.0134592 (PMC4552844; doi:10.1371/journal.pone.0134592)
Supplement: S3 Table — (DOCX) [file pone.0134592.s004.docx]

**S3 Table. Assembly metrics for sequencing libraries from typical CC97 and ST71-like isolates.**

| **Isolate name** | **MOK009** | **MOK018** | **MOK023** | **MOK029** | **MOK032** | **MOK042** | **MOK063** | **MOK099** |
| --- | --- | --- | --- | --- | --- | --- | --- | --- |
| **Clonal complex** | 97 | 97 | 97 | 97 | 97 | 97 | 97 | 97 |
| **Sub-group** | ST71-like | Typical CC97 | Typical CC97 | Typical CC97 | Typical CC97 | ST71-like | Typical CC97 | ST71-like |
| **No. of scaffolds** | 23 | 31 | 22 | 70 | 20 | 31 | 19 | 20 |
| **Sum of bp** | 2,713,655 | 2,860,941 | 2,852,263 | 2,899,666 | 2,851,707 | 2,872,377 | 2,782,623 | 2,723,059 |
| **Min scaffold size** | 636 | 612 | 648 | 606 | 749 | 599 | 649 | 685 |
| **Max scaffold size** | 538,020 | 656,150 | 657,205 | 656,150 | 881,559 | 834,409 | 556,216 | 823,519 |
| **Average scaffold size** | 117,985 | 92,288 | 129,648 | 41,423 | 142,585 | 92,657 | 146,453 | 136,152 |
| **N50** | 452,238 | 417,120 | 413,310 | 413,264 | 579,500 | 523,929 | 437,650 | 370,977 |
| **No. of open reading frames** | 2,524 | 2,727 | 2,722 | 2,754 | 2,723 | 2,746 | 2,617 | 2,561 |
